# Control methodology of synchronous lifting for the dual forging manipulator at clamping condition

**DOI:** 10.1038/s41598-022-07220-5

**Published:** 2022-03-10

**Authors:** Fugang Zhai, Zhiqiang He, Yanfeng Zhao, Liu Yang, Xiangdong Kong

**Affiliations:** 1 School of Mechanical Engineering, Yanshan University, Qinhuangdao, 066004 China; 2 Hebei Innovation Center for Equipment Lightweight Design and Manufacturing, Qinhuangdao, 066004 China

**Keywords:** Engineering, Mechanical engineering

## Abstract

Asynchronous coupling force of dual forging manipulator frequently results in poor forging and even equipment failure. In this paper, a synchronous control strategy in dual forging manipulator systems (DFMS) is proposed to stabilize its operation. Kinematic model of the hanging system and finite element model of the forgings are established to investigate the relationships of tension, forging deformation and deformation rate. The rigid-flexible coupling model of DFMS is further established and simulated concerning hydraulics, mechanics and controls. A correction based on the independent feedback state difference is concerned, simulated results show good agreements with experimental data, validating the dead zone compensation algorithm of the proportional valve. Moreover, by the control strategy, the vertical synchronous error of the pincers end is rather small as ± 0.125 mm. The methodology presented in this paper represents a fundamental step towards the cooperation of DFMS and the press to realize collaborative operations.

## Introduction

**Figure 1:**
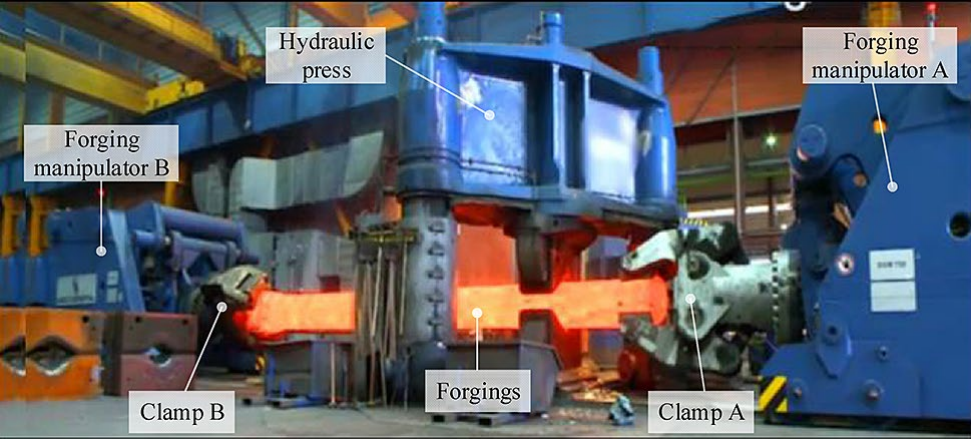
DDS released a double forging manipulator operating the example.

Nowadays, with the booming development of the manufacturing industry, there is an increasingly strong demand for high-quality large-scale forgings. As one of the important pillars in the manufacturing field, the improvement of equipment performance is crucial to the development of the manufacturing industry. Especially the forging manipulator is a piece of important auxiliary equipment in the field of mechanical processing, which significantly improves the forging efficiency and quality^1^. In particular, forging manipulator is important auxiliary in the machining field, whose competence represents the national forging level. Normally, on one hand, the forging manipulator can be classified into the single-mode and the dual one. The latter mode can accurately adjust the forging state by two synchronous clamps, especially suitable for long alloy forgings with a narrow temperature range^2–4^. On the other hand, rotation, translation and lift are three main motions during the whole forging process. Because of the inherent inconsistency between the two hydraulic systems driving clamping, the synchronous lifting control method that is of great significance to stabilize the forging process^5^. However, so far, few related articles concern the synchronous clamping lifting control methodology in the DFMS and its function has not been recognized. Figure 1 is the example of the double forging manipulator first released by The German DDS in 2018. It consists of a press and two forging manipulators A and B placed relative to each other. The clamps in the front of the two manipulators clamp both ends of the forgings at the same time. During the forging process of the press, the manipulators A and B adjust the posture and move the forgings to complete the forging operation with the press. But in the drawing process, the technique and experimental data of complex and urgent control problems have not been published.

Zhao et al.^6^ obtained approximate analytical solutions of nonlinear equations of nanobeam considering its surface effects by using the Galerkin and complex normal form methods, and analyzed the influence of residual surface stress on the nonlinear dynamic behavior of Nano/Micro-electro-mechanical system. Guan et al.^7^ established an inverse dynamics model of a similar model of forging manipulator and solved the nonlinear dynamics problem of the controller by the principle of virtual work feedforward and feedback linearization methods. Wang et al.^8^ proposed a hybrid control strategy of pressure and position, realizing their mutual switches in different buffer stages. Li et al.^9^ proposed a force compliance method with pressure and verified it with co-simulation technologyz. Liu et al.^10^ investigated the synchronization control of a double hydraulic cylinder, which is controlled directly by a high-speed on–off valve, to solve the problem of a hydraulic synchronization system. Collaborative synchronization control algorithm and PWM-PFM are validated by simulation and experiment. Sturov et al.^11^ adopted the trajectory synchronization method, so that the gas–liquid energy storage system can support the synchronization frequency of low-frequency grid events. Ding et al.^12^ designed an approximate internal model controller for the non-affine nonlinear subsystems to realize velocity trajectory tracking control, and combined this method with the position feedback control based on the recursive design idea to achieve position trajectory tracking control. Admittedly, the above researches mainly relate to the synchronous control method of single forging manipulators and hydraulic systems. These achievements provide new insights into the synchronous lifting control in DFMS.

**Figure 2:**
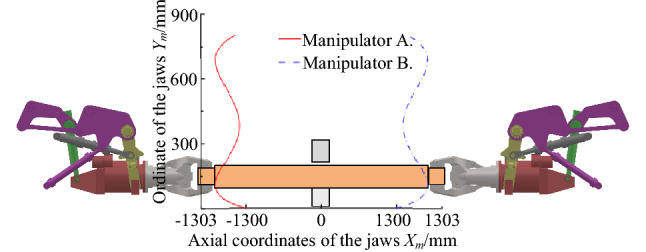
The movement track of two forging manipulators.

Control system is much more complicated in dual operating machine**s** compared with single modes. More specifically,

1. Nonlinear moving trajectory of the clamp is caused by the hanging device coupling. The feature of trajectory is an “S” curve, as shown in Fig. 2.
2. Strong plasticity in high temperature forging conditions. In this case, plastic deformation frequently arises by external force in DFMS.
3. Weak rigid connection and slow time-dependent change between the clamps and the high-temperature forging. When the force imposed on the forging is larger than the friction, the forging may slide or even drop from the jaws^13^.

All these issues justify further studies in the coordination and synchronous control method between two manipulators. Because of the coupling problem of the hanging mechanism configuration of the forging manipulator itself, the track of the clamping end shows the rule of "S", and the lateral displacement generated by the track of "S" causes the phenomenon of "dragging" in the process of lifting, which causes forging deformation and coming off the clamp. Moreover, due to many degrees of freedom of hanging devices and their interaction, coupled axial displacements of two clamps will arise simultaneously under bad coordination. Additionally, with high load, large inertia and strong nonlinearity of clamping systems, a large synchronous coupling force frequently occurs during the lifting process, degrading the forging quality or even damaging the equipment^14^. From the perspective of economy and anti-pollution, a common proportional valve is usually adopted in the position closed-loop of the hanging system to control a single rod in a hydraulic cylinder. Meanwhile, to decrease both processing cost and neutral leakage, the proportional directional valve with the positive overlap in the valve core is used. However, this structure often causes a larger dead zone, increasing the steady-state error and following error of the proportional closed-loop system, and further threatening system stability^15^. Thus, to mitigate the influence of the dead zone on the position closed-loop system, a related compensation algorithm is necessary.

In this paper, the principle of mechanics and the hydraulic system of the DFMS are discussed and analyzed in the cooperative lifting condition. By introducing a dead zone compensation algorithm and taking coupling effect during lifting into account, a rigid-flexible coupling model of DFMS is further established and simulated concerning hydraulics, mechanics and controls. A synchronous control methodology is proposed to reduce the coupling force when the DFMS performs the forging lifting, improve the forming quality, and protect equipment from damage.

## Principle analyses

### Principle of the dual forging manipulator

**Figure 3:**
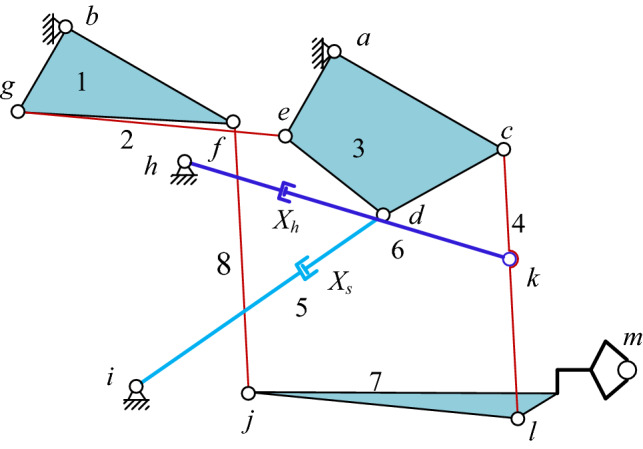
Kinematic sketch of suspension system of the DFMS.

The hanging system, the core of the manipulator, mainly helps to adjust the clamp position and mitigates the deformation resistance of the forging. Its kinematics greatly affects the forging dimensional accuracy and the processing quality. Herein, attention is given to the 20 kN rail hydraulic DFMS in the "5 MN fast forging hydraulic unit test rig" at Yanshan University. The two suspension systems in the DFMS have the identical mechanical principle, namely the parallel connecting type^16^. The corresponding structural sketch is illustrated in Fig. 3 Mass of each component and their moment of inertia to the mass center is shown in Table 1. The buffer cylinder *X*_*h*_ in Fig. 3 mainly works to cushion the deformation resistance of forgings, and the lifting cylinder *X*_*s*_ is used to adjust the angle of the forgings with the horizontal direction. Additionally, marks 1–7 are the components of the suspending system, *a* ~ *k* are the related hinge points, m notates the end of the clamp. The filling regions 1, 3, and 7 are equivalent to linkages with constant size.

**Table 1:** Main kinematic parameters of the suspension system.

Components	Mass/kg	Moment of inertia to the center of mass /kg m^2^
1	360.9	38.68
2	86.7	10.48
3	475.6	59.30
4	679	71.02
5	241.6	241.6
6	373.4	373.4
7	2848.4	2848.4
8	339.8	339.8

### Principle of manipulator hydraulic system

**Figure 4:**
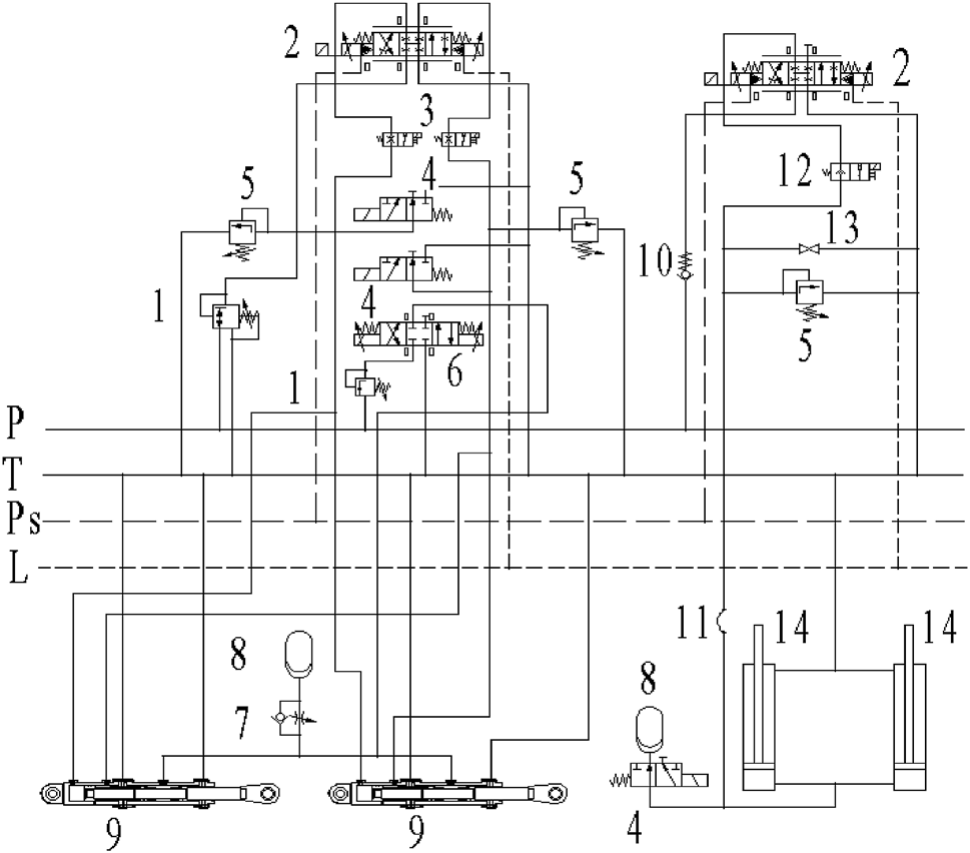
Hydraulic schematic diagram of suspension system of manipulator**s** A and B (1. relief valve; 2. high frequency proportional directional valve; 3. electromagnetic ball valve 1; 4. two-position three-way solenoid directional valve; 5. overflow valve; 6. three-position four-way solenoid directional valve; 7. one-way throttle valve; 8. accumulator; 9. cushion cylinder; 10. check valve; 11. expansion joints; 12. electromagnetic ball valve 2; 13. stop valve; 14. lifting cylinder).

The dual forging manipulators marked as A and B in this paper are both driven by constant pressure variable pump and accumulator. The corresponding hydraulic system adopts a centralized oil supply scheme, which can be approximated as a constant pressure source. The lifting and buffering operations of manipulators A and B are driven by valve-controlled hydraulic cylinders, with the identical hydraulic principle. It is inferred that the buffer circuit has both the active and the passive control function to meet the requirements of the subsequent test and debugging needs. The hydraulic scheme of the suspension system of manipulators A and B is shown in Fig. 4.

In the figure above, *P* denotes the high-pressure oil port, *T* is the oil return port, *P*_*s*_ is the control oil port, and *L* is the control port of oil return. The main parameters of the hydraulic system are shown in Table 2.

**Table 2:** Main parameters of the hydraulic system.

Symbol	Parameter	Value	Unit
*P*_*s*_	System pressure	2 × 10^7^	Pa
*P*_*T*_	Return pressure	3 × 10^5^	Pa
*β*_*e*_	Oil bulk modulus	1.5 × 10^9^	Pa
*ρ*	Oil density	878	kg/m^3^
*w*_*svl*_	The natural frequency of proportional valve of manipulator A	345	rad/s
*ζ*_*svl*_	The damping ratio of the proportional valve of manipulator A	0.84	–
*w*_*sv2*_	The natural frequency of proportional valve of manipulator B	100	rad/s
*ζ*_*sv2*_	The damping ratio of the proportional valve of manipulator B	0.86	–
*δ*_*1*_	The dead zone of the proportional valve of manipulator A	0	–
*δ*_*2*_	The dead zone of the proportional valve of manipulator B	0.15	–

A better understanding of the mechanism and hydraulic principle of the suspension system will facilitate the establishment of the relevant simulation model.

## Simulation model establishment

### Numerical simulation of forgings under axial squeezing conditions

The shape of the forging is variable and its deformation is hard to mathematically describe under axial squeezing or stretching conditions. Therefore, the elastic–plastic method is used to simulate the forging to reveal the relationship of deformation resistance, deformation, and deformation rate. Because the axial deformation of the forging is relatively small, the stress–strain relationship keeps the same under both squeezing and stretching conditions. Therefore, this study only simulates the process of axial extrusion by the clamps in DFMS.

**Figure 5:**
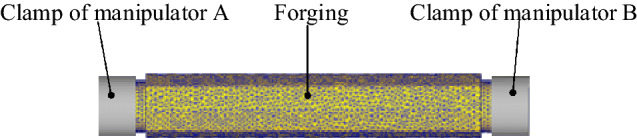
Axial squeezing model of the forging.

The finite element simulation software is used. The dimension of the forging main part is *Φ*600 × 2150 mm, the clamping part is *Φ*300 × 300 mm. The material of the forging is 35CrMo with a density of 7850 kg/m^3^. The forging is set as elastic–plastic solid with a temperature of 1000 °C There is no sliding between the clamps and the forging so a rigid connection is hypothesized between them. Three-dimensional models of the forging are shown in Fig. 5.

**Figure 6:**
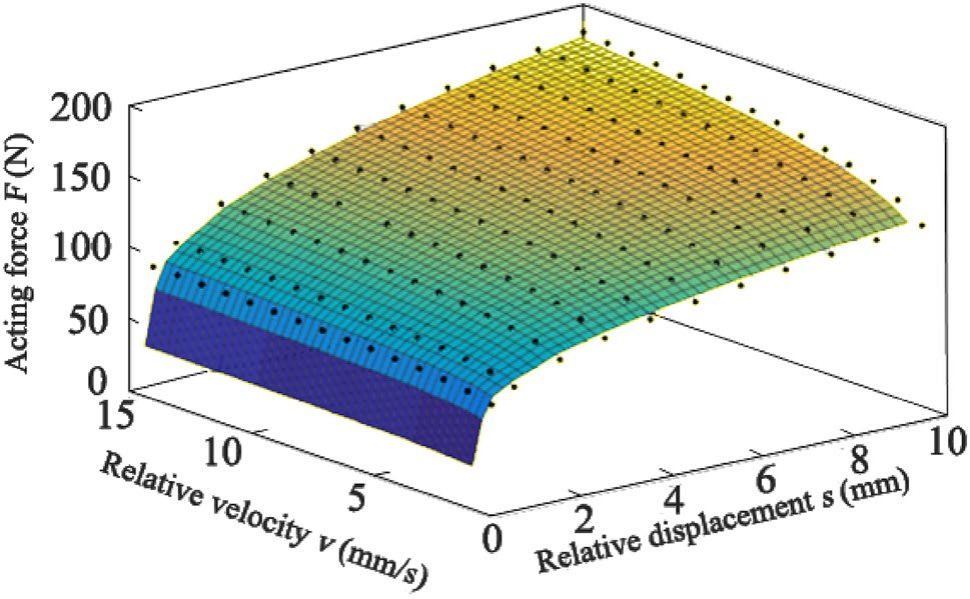
The axial load of the clamp against displacement *s* and velocity *v*.

In this simulation scheme, the axial squeezing phenomenon is realized by the asynchronous motion of the two manipulators. More specifically, the manipulator A is stationary, and the clamps of the B axially move at a certain speed *v* against the forging until the displacement *s*. The reactive force F on clamp B imposed by the forging is simulated at different relative speeds. Results are located in Fig. 6 by the black dots. Linear interpolation is used to obtain the axial load distribution against displacement and velocity of the clamp. A positive proportional relationship is revealed in Fig. 6. That is, the force on the clamp ascends with the increase of *s* and *v*, gradually approaching the peak of the surface.

### Modelling of DFMS under synchronous lifting condition

**Figure 7:**
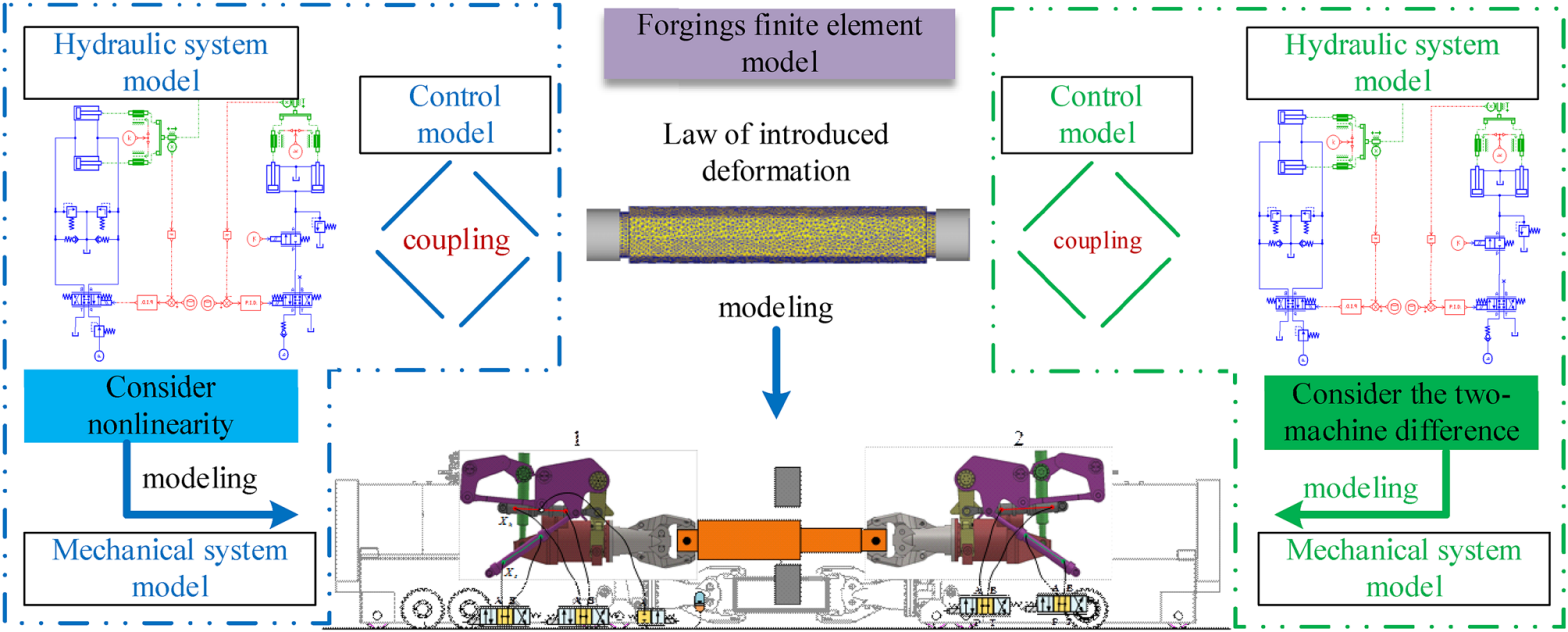
Simulation model of dual forging manipulator’s synchronous lifting.

A rigid-flexible coupling collaborative model of the DFMS is established by using the complex system modeling and simulation platform, as seen in Fig. 7. Components concerning this simulation model include hydraulics, mechanics, controls and forgings. Two suspension systems are connected by the forging. In Fig. 7, the blue and the green block respectively include the mechanical module, hydraulic module and control module of manipulators A and B. These two components are connected by a rigidity-flexible coupling collaborative model of the forging. Specific modeling methods are described in the literature^17–19^.

## Synchronous control strategy of the DFMS lifting cylinder

### Dead zone compensation algorithm of valve-control cylinder

The control performance of the electro-hydraulic proportional system attributes to both the external load and the component characteristics. Considering investment and anti-pollution ability, a common proportional valve and a cylinder with a single rod are usually used to complete the position closed-loop system. This scheme can often cause a low-frequency response of the system, deteriorating the control performance^20^. To reduce the processing cost and the neutral leakage, the core of the proportional directional valve generally adopts a positive overlap structure, so that this valve has a large dead zone. As a result, steady-state error and tracking error of the closed-loop system increase, degrading the system stability. Hu et al. proposed a robust nonlinear integrated adaptive algorithm with unknown nonlinear dynamic systems for control schemes of asymmetric and uneven slopes in dead zones^21^. Oh, et al. introduced an adaptive fuzzy logic controller to solve the problem of dead zone dependent inaccuracy in time-varying nonlinear systems. However, the proposed controller is only applicable to symmetric deadband nonlinearity^22^. Wang et al.^23^proposed an integrated control strategy of position synchronization control for dual-electro-hydraulic actuators with unknown dead-zones, the model of the electro-hydraulic proportional position control system (EPPS) was identified by the forgetting factor recursive least square (FFRLS) algorithm, the position control accuracy of each hydraulic actuator is guaranteed. Tan et al.^24^ proposed a recursive method to identify dead zones between two systems. However, this approach is limited to light-duty systems. Here, the two manipulators use a common proportional valve with a dead zone of 15%. Therefore, the dead zone must be compensated by the algorithm to alleviate the effects.

The smallest dead zone range is generally taken as the compensation value to ensure a better stable system. The dead zone compensation algorithm can be expressed in Eq. (1), where *δ*_*1*_**,** *δ*_*2*_ are separately the upper and lower limit of the valve dead zone threshold, *δ*_*3*_ is the reserved dead zone threshold, *u*_*3*_ is the saturation voltage input by the proportional valve, *u*_*g*_ is the output voltage after compensation, *u*_*1*_ is the input voltage before compensation.

1 $$u_{g} = \left\{ {\begin{array}{*{20}c} {u_{1} + \delta_{1} u_{m} } & {\delta_{3} u_{m} < u_{1} } \\ {\frac{{\delta_{1} }}{{\delta_{3} }}u_{1} } & {0 < \, u_{1} \le \delta_{3} u_{m} } \\ {\frac{{\delta_{2} }}{{\delta_{3} }}u_{1} } & { - \delta_{3} u_{m} \le u_{1} \le 0} \\ {u_{1} - \delta_{2} u_{m} } & { \, u_{1} < - \delta_{3} u_{m} } \\ \end{array} } \right.$$

**Figure 8:**
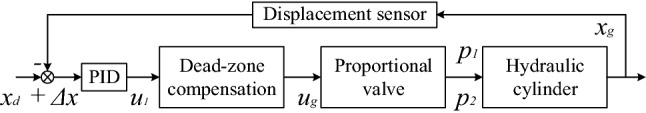
Diagram of proportional closed-loop position control with dead-zone compensation.

The principle of dead zone compensation is based on the PID control strategy. *u*_*g*_ is determined according to the *u*_*1*_ value output by the PID controller. Notably, the reserved dead zone *δ*_*3*_ will inevitably trigger additional errors in the system. The value of *δ*_*3*_ can be set according to the accuracy requirement. The block diagram of proportional closed-loop position control with dead-zone compensation is shown in Fig. 8. Its principle is that the PID controller output *u*_1_ signal results from the displacement error Δ*x* of the valve controlled cylinder, and determines *u*_*g*_ signal to adjust the opening of the valve port through the dead zone compensation algorithm. And then the output flow and the displacement of the valve controlled cylinder, are changed and adjusted accordingly. Hence, a position closed-loop system is formed.

**Figure 9:**
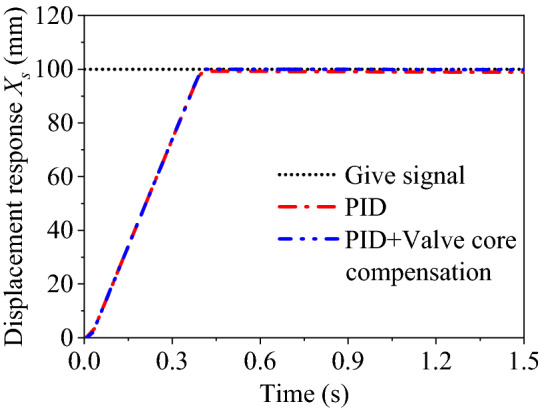
Displacement response comparison of manipulator B.

Based on this compensation algorithm, the position closed-loop system of the lifting cylinder B is simulated and analyzed. Given a step signal of 100 mm, the PID controller parameter is set to *P* = 2. The displacement response curves without and with compensation are respectively shown in Fig. 9.

Results in Fig. 9 justify the effectiveness of the compensation algorithm compared with the traditional PID controller. And the steady-state error can be reduced from 1.5 to 0.06 mm approximately.

### Synchronous control strategy for equivalent cross-coupling correction

The synchronous movement of DFMS lifting operation is often determined by multiple actuators. Therefore, a synchronous control strategy is needed to coordinate the synchronous control of the hoist cylinder. At present, the synchronization control strategy is widely used Xu et al. proposed a synchronization control strategy of combining cross-coupling errors in multi-layer and multi-axis systems, and the motor synchronous error is low^25^. Zhao et al.^26^ designed the hydraulic control system of the dual-cylinder synchronous motion, and factors affecting synchronous performance are analyzed. To achieve synchronization performance with robustness, Zhao et al. designed a cross-coupled synchronous system based on the virtual electronic shaft in which both torque and speed are used as feedback signals are developed^27^. Han et al.^28^ to achieve higher precision in controlling three hydraulic cylinders, set up an electro-hydraulic position control system of water hydraulic manipulator in the process of CFETR blanket maintenance by using AMESim, this scheme can guarantee stability and improve the operational performance to a certain extent. According to the simulation model in Fig. 7 and the system parameters in Tables 1 and 2, a synchronous control strategy is proposed to minimize the position error between the two cylinders. And the following characteristics of their trajectories are studied.

**Figure 10:**
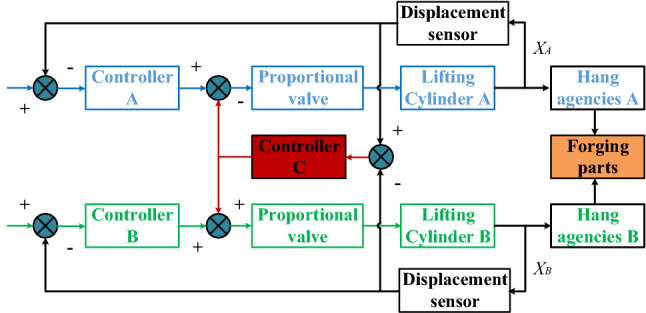
Block diagram of synchronous control with equivalent cross-coupling correction.

The block diagram of the DFMS synchronous control based on independent feedback state difference correction is shown in Fig. 10. Displacement sensors detect the movement of cylinders A and B to calculate the synchronous error. The correction signal is obtained by putting this error into controller C, and then is fed back to the outlet of the controller the A and the B. The lifting cylinder A is controlled to follow cylinder B and finally to approach a synchronous state.

### Simulation results of the synchronous control of the lifting cylinders of the dual manipulators

**Figure 11:**
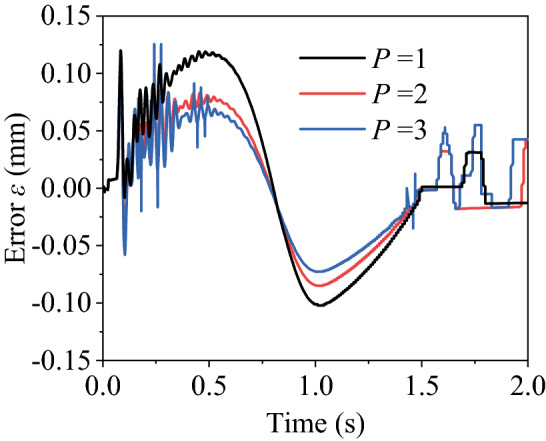
Curves of synchronous error.

PID parameters of controller C in Fig. 10 vary as *P* = 1, 2, 3 while those of the controllers A and B are kept constant. In such cases, synchronous errors of the two cylinders are shown in Fig. 11.

With the increase of parameter *P*, the curve oscillates more obviously during the initial segment. But the subsequent synchronization error will be smaller. The maximum and minimum errors against *P* are listed in Table 3.

**Table 3:** Synchronous error under different values of *P*.

Items	1	2	3
Maximum (mm)	0.1115	0.103	0.127
Minimum (mm)	− 0.102	− 0.073	− 0.066

**Figure 12:**
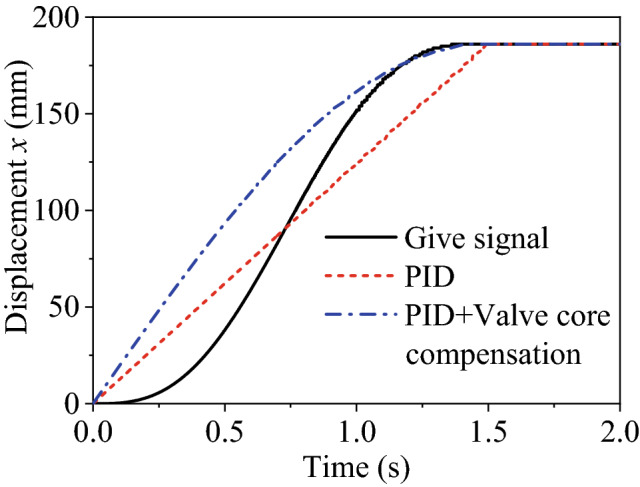
Moving trajectories of the lifting cylinder.

Three moving patterns are designated to the lifting cylinders, namely the ramp, sine and the preceding planning curve. The rise time of the three curves is 1.5 s, and the stroke is 186.5 mm. Their trajectories are illustrated in Fig. 12.

**Figure 13:**
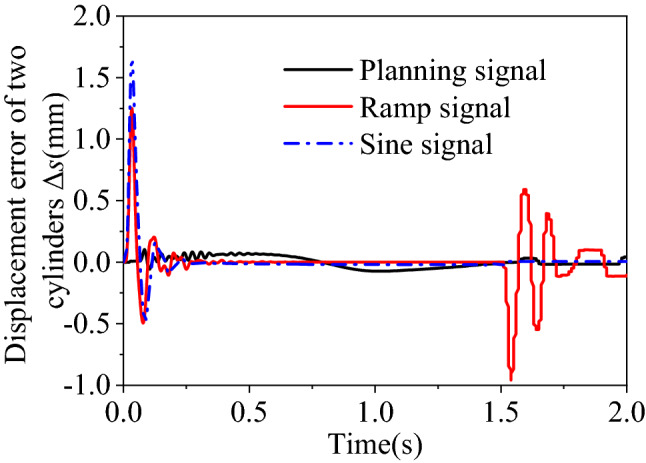
The synchronous error of lifting cylinder.

Three curves in Fig. 12 are input as given signals to control the DFMS. With the synchronous strategy of equivalent cross-coupling correction and *P* = 2 for controller C, synchronous errors of these three schemes are shown in Fig. 13.

It is evident that the synchronous error changes a lot to concerning the designated signals. The exact value of the error is tabulated in Table 4.

**Table 4:** Synchronous error under different signals.

Signal types	Ramp signal	Sine signal	Planning signal
Maximum (mm)	1.208	1.70	0.103
Minimum (mm)	− 0.94	− 0.496	− 0.073

The whole lifting process includes the startup phase, the intermediate phase and the stop phase. They are separately analyzed. Related results are as follows:

1. During the startup phase, the initial speed of signal Sine is the largest, and is lower for the Ramp. The initial speed of the planning signal is zero while a sudden slope occurs for the other two signals. This saltation on the displacement curve induces a larger synchronous error for the two valve-controlled cylinders. And this error is proportional to the startup speed.
2. During the intermediate stage, the error under the Planning signal is greater than those of the other two signals since its more obvious speed change is in Fig. 12.
3. During the stop phase, the error curve drastically fluctuates only for the Ramp signal. This attributes to the that speed of the Ramp signal suddenly changing from a constant value to 0 in Fig. 12.

So the speed variation of motion trajectory justifies careful design to ensure a stable and synchronous lifting movement. Attention should be paid to avoid sudden speed changes.

## Experimental validation of dead-zone test and compensation algorithm

**Figure 14:**
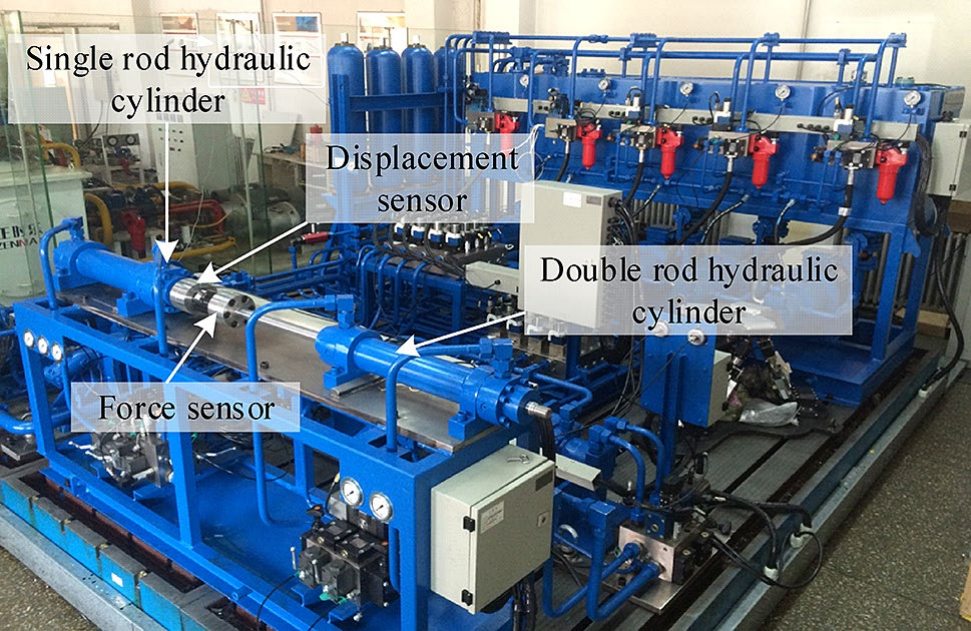
Experimental platform.

**Figure 15:**
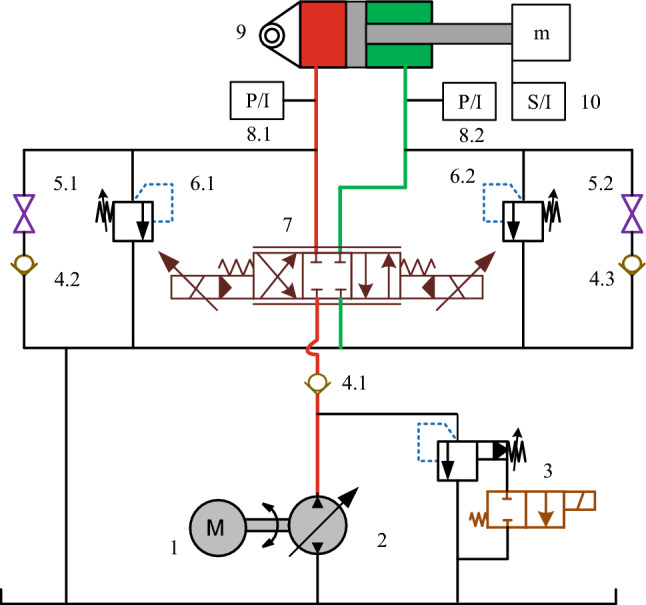
Schematic diagram of test rig hydraulic system (1. motor; 2. pump; 3. electromagnetic overflow valve; 4. check valve; 5. stop valve; 6. safety valve; 7. proportional direction valve; 8. pressure sensor; 9. double rod hydraulic cylinder; 10. displacement sensor).

This validation is conducted by the electro-hydraulic servo system test rig at Yanshan University. Figure 14 presents components of the experimental device and its hydraulic system is shown in Fig. 15.

Herein, the control valve 7 is a three-position four-way high-frequency proportional valve with the control signal of − 10 ~ 10 V. The stroke of the cylinder 9 is 400 mm, and its diameter is 80 mm. The diameter of the rod is 36 mm.

**Figure 16:**
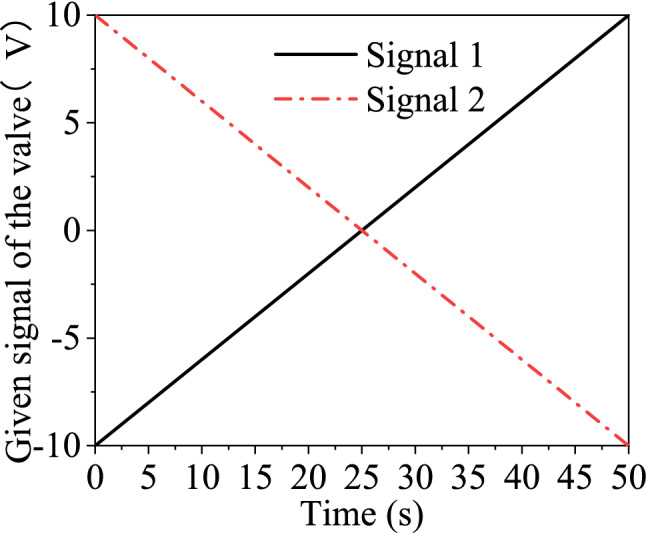
Signals were dispatched to the proportional valve.

The dead zone of the hydraulic system is detected first to further validate the compensation algorithm. While the system pressure is 5 MPa, parameter *P* of the PID controller is set to be one. An open-loop test is performed on the valve-controlled hydraulic cylinder. Two different ramp signals shown in Fig. 16 are input to the proportional valve.

**Figure 17:**
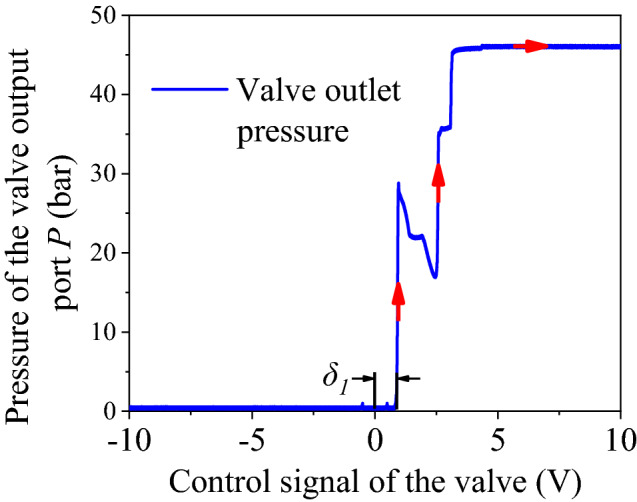
Pressure at the valve outlet (signal 1).

**Figure 18:**
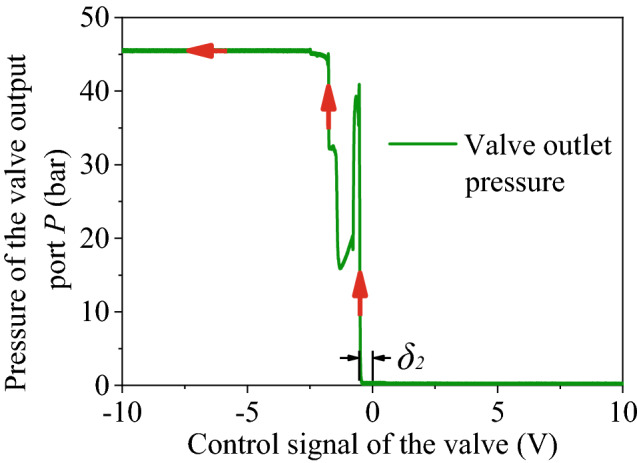
Pressure at the valve outlet (signal 2).

As for signal 1, the hydraulic cylinder first retracts and then extends. Pressure at the valve outlet is shown in Fig. 17. On the contrary, the cylinder first extends and then retracts for signal 2. The corresponding pressure curve at the outlet is shown in Fig. 18.

It can be seen from Fig. 17 that when the input signal locates in the range of − 10 ~ 0.8 V, the output port is connected to the fuel tank. Pressure at the value outlet is zero, and no cylinder extends. When the control signal exceeds the valve’s dead-zone upper limit of 0.8 V, a sudden pressure climb occurs. When the pressure increases enough to overcome the static friction, the cylinder starts to move, and the pressure will drop transitorily. When the oil cylinder is about to be completely extended, the pressure will suddenly climb to about 35 bar due to the cushioning structure at the end. At the same time, the oil cylinder decelerates and ultimately reaches the end, where the pressure is 46 bar. Similarly, it can be seen from Fig. 18 that the valve’s dead-zone lower limit is 0.5 V.

**Figure 19:**
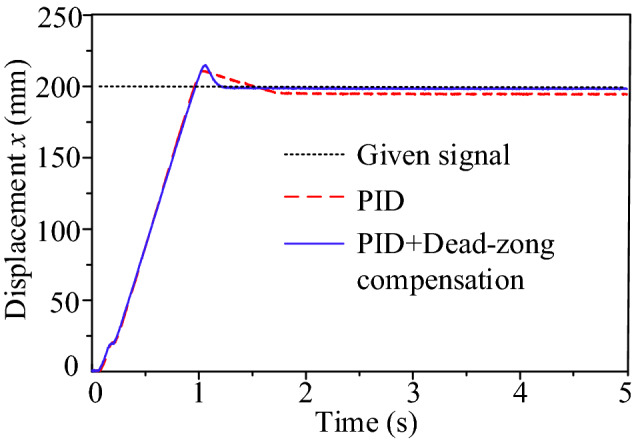
Displacement response curve.

Based on the calculated dead zone value, the position closed-loop response of the valve-controlled hydraulic cylinder is analyzed and compared with and without compensation algorithm. And the displacement response to a 200 mm step signal is presented in Fig. 19 while the controller PID parameter *P* = 0.04.

Two resultant curves show overshoot though a small proportional gain. This may attribute to the test under no-load conditions. In terms of rapidity, the rise time is about 1 s, and the adjustment time with only PID is longer, around 1.7 s. Concerning accuracy, the steady-state error for only PID is up to 6 mm, while is only about 0.5 mm for PID + dead zone compensation. These comparisons justify that the dead-zone compensation algorithm is effective.

## Conclusions

This paper analyzes the mechanic and hydraulic principles of the dual forging manipulator. A dead-zone compensation algorithm is proposed for the proportional valve and is validated by simulation and experiment. Meanwhile, the forging database is formed by simulating the forging clamping. Then a rigid-flexible coupling collaborative simulation model of the dual forging manipulator is established concerning hydraulic, mechanical, control and forging.

Based on the established coupling collaborative simulation model and the independent feedback state difference correction, an equivalent cross-coupling correction synchronous control strategy is proposed. The simulated synchronous error at the end of two clamps in the vertical direction is within ± 0.125 mm. And various synchronous errors are analyzed when the lifting cylinder moves through different patterns. Achievements in this paper represent a promising step towards the design of cylinder lifting trajectory and to the synchronous control of dual manipulator.

## Data Availability

All data generated or analysed during this study are included in this published article.
